# Identification and Characterization of an Intergenic “Safe Haven” Region in Human Fungal Pathogen *Cryptococcus gattii*

**DOI:** 10.3390/jof8020178

**Published:** 2022-02-11

**Authors:** Yeqi Li, Tuyetnhu Pham, Xiaofeng Xie, Xiaorong Lin

**Affiliations:** 1 Department of Microbiology, University of Georgia, Athens, GA 30602, USA; Yeqi.Li@uga.edu (Y.L.); xiaofeng.xie@uga.edu (X.X.); 2 Department of Plant Biology, University of Georgia, Athens, GA 30602, USA; nhu.pham@uga.edu

**Keywords:** *Cryptococcus gattii*, safe haven, ectopic integration, genome editing, complementation

## Abstract

*Cryptococcus gattii* is a primary fungal pathogen, which causes pulmonary and brain infections in healthy as well as immunocompromised individuals. Genetic manipulations in this pathogen are widely employed to study its biology and pathogenesis, and require integration of foreign DNA into the genome. Thus, identification of gene free regions where integrated foreign DNA can be expressed without influencing, or being influenced by, nearby genes would be extremely valuable. To achieve this goal, we examined publicly available genomes and transcriptomes of *C. gattii*, and identified two intergenic regions in the reference strain R265 as potential “safe haven” regions, named as CgSH1 and CgSH2. We found that insertion of a fluorescent reporter gene and a selection marker at these two intergenic regions did not affect the expression of their neighboring genes and were also expressed efficiently, as expected. Furthermore, DNA integration at CgSH1 or CgSH2 had no apparent effect on the growth of *C. gattii*, its response to various stresses, or phagocytosis by macrophages. Thus, the identified safe haven regions in *C. gattii* provide an effective tool for researchers to reduce variation and increase reproducibility in genetic experiments.

## 1. Introduction

Cryptococcosis caused by the pathogenic *Cryptococcus* species complexes is responsible for 181,100 deaths annually [1]. The majority of cryptococcosis cases in immunocompromised individuals such as AIDS patients and organ transplant recipients are caused by the *Cryptococcus neoformans* species complex [2,3,4]. By contrast, the related *C. gattii* species complex is a primary pathogen and can cause fatal infections in both immunocompromised individuals and individuals with no apparent immunodeficiencies [5,6]. Although both *C. neoformans* and *C. gattii* cause fatal meningitis, *C. gattii* is more likely to cause severe lung disease and death, without CNS dissemination [7]. *C. gattii* is responsible for the Vancouver Island outbreak in 1999, and it has spread to the north–western US [8,9]. *C. gattii* diverged from *C. neoformans* roughly 37 million years ago [10]. The *C. gattii* species complex comprises four distinct molecular types, VGI-VGIV [6].

In the past two decades, major advances in molecular technology have facilitated our understanding of the pathogenic *Cryptococcus* species complexes [11,12,13,14]. Classic approaches such as gene complementation, gene overexpression, and construction of fluorescent tagged protein strains are routinely used to analyze gene functions and all require introduction of foreign DNA into the genome [15,16]. This is often achieved by ectopic integration, which can cause varied expression levels of the integrated genes due to position effects, or unintended disruption of gene(s) near the integration site [17,18]. Thus, integration in a designated gene free region in the genome is preferred. Several such gene free regions named “safe haven” sites have recently been identified in two reference strains of the *C. neoformans* species complex (H99 and XL280), and Af293/CEA10 strains of *A. fumigatus* [18,19,20,21]. The identification of these safe haven sites has greatly facilitated genetic research in these fungal species [22,23,24,25]. However, such safe haven sites have not been identified in *C. gattii*.

In this study, we took advantage of the genomic data and transcriptome data that are publicly available for *C. gattii* to facilitate the identification of potential gene free regions that are unlikely to be in a constitutive heterochromatin state. We identified two such gene free regions as potential “safe haven” sites: CgSH1 and CgSH2. We found that integration of a fluorescent and a selection marker gene at CgSH1 and CgSH2 did not affect the expression of their neighboring genes. Moreover, DNA integration at these regions *per se* had no apparent effect on all the phenotypes examined, including growth, thermotolerance, melanization, capsulation, tolerance of cell wall stress, response to osmotic stresses, and phagocytosis. Therefore, we believe that CgSH1 and CgSH2 provide safe haven sites to facilitate genetic manipulations in *C. gattii* strains. Together with the previous work for the *C. neoformans* species complex, this study completes the important task of identifying safe haven regions for the entire pathogenic cryptococcal species complexes.

## 2. Materials and Methods

### 2.1. Procedures to Identify Potential Safe Haven Sites

The genomic sequence and annotation files of *C. gattii* R265 were downloaded from NCBI (GenBank assembly accession: GCA_002954075.1). According to the orientation and positions of two neighboring genes, we classified the intergenic regions as convergent, divergent, or tandem and calculated their sizes. BAM (binary alignment map) files and FPKM values were generated from published RNA-seq data [26] using Trim_Galore (0.6.5), STAR (2.7.1a) and Cufflink (2.2.1). BAM files were visualized with Integrative Genomic Viewer (2.6.3) to check the transcript of the intergenic regions and neighboring genes.

To identify the potential CgSH1 in other *C. gattii* strains, a BLAST search was conducted using the sequence of CNBG_3433 as a query to identify homologues in other *C. gattii* strains. Then we performed subsequent BLAST analyses using the sequences of neighboring genes of the homologues as queries, and we then identified the best match for CNBG_3434 in these strains. Although CNBG_3433-CNBG_3434 and their homologue pairs remain neighboring genes in other *C. gattii* strains examined, the orientation of these two neighboring genes is not conserved among the 6 strains. The same strategy was used for identifying potential CgSH2 in other strains of *C. gattii*.

### 2.2. Strains and Media

*Cryptococcus gattii* strains and plasmids used in this study are listed in the Supplemental Table S1. All strains were cultured in YPD (1% yeast extract, 2% peptone, 2% dextrose and 2% agar) at 30 °C unless stated otherwise. RPMI (catalogue no. SH30011.04, Cytiva), YNB (0.67% yeast nitrogen base with no amino acids, 2% dextrose, 2% agar), V8 (5% V8 juice, 0.05% KH_2_PO_4_, 4% agar, pH 7), and L-DOPA medium (15 mM dextrose, 10 mM MgSO_4_, 29.4 mM KH_2_PO_4_, 13 mM glycine, 3 µM thia-mine, and 1 mM L-DOPA, pH 5.5) were used to test the phenotypes as described previously [27,28]. Strains were stored as 15% glycerol stocks at −80 °C.

### 2.3. Generation of mNeonGreen Strains

To generate mNeonGreen fluorescence strains, we used the Transient CRISPR-Cas9 Coupled with Electroporation (TRACE) system, as described previously [29]. Firstly, we amplified the mNeonGreen expression construct with a hygromycin (HYG) drug selection marker from plasmid pYZ25 using M13F and M13R primers. The amplified construct was used as the donor DNA. To generate the sgRNA construct, we designed sgRNA target sequence using the Eukaryotic Pathogen CRISPR guide RNA/DNA Design Tool (http://grna.ctegd.uga.edu/, accessed on 21 January 2022) embedded in FungiDB. *Cryptococcus* native U6 promoter was amplified from JEC21 while the scaffold of sgRNA was amplified from the plasmid pDD162 [30]. Then the U6 promoter, 20-nt guide sequence, scaffold, and 6-T terminator were assembled using single-joint PCR. For transformation, we needed transient Cas9 expression driven by the highly active *TEF1* promoter. To that end, the plasmid pXL-Cas9 with the *GPD1* promoter driven Cas9 was digested with *Not*I and *Fse*I and then purified as the backbone vector. The *TEF1* promoter was amplified from pYZ79 and purified [31]. After T4 ligation of these two digested products, the plasmid carrying P*_TEF1_*-Cas9 was generated and used as the template to amplify the whole Cas9 expression cassette with primers M13F/M13R [13]. Three DNA fragments—donor DNA, sgRNA, and P*_TEF1_*-Cas9—were introduced into R265 by electroporation and transformants were selected on YPD plates with hygromycin (200 µg/mL) and passaged on non-selective YPD media for stability testing. Transformants with correct integration into the CgSH1 were expected to yield a band of ~1.2 kb for the forward orientation and ~1 kb for the reverse orientation with the primer set Linlab3779/Lianlab8147/Linlab8148 (Figure S1). Transformants with correct integration into CgSH2 were expected to yield a band of ~1.2 kb for the forward orientation and ~1 kb for the reverse orientation with the primer set Linlab3779/Lianlab8152/Linlab8153 (Figure S1).

### 2.4. Quantitative Real-Time PCR

The indicated strains were cultured in liquid YPD medium at 30 °C with shaking at 220 rpm for 16 h. Cells were collected and flash frozen in liquid nitrogen, and then lyophilized for 24 h. Total RNA samples were extracted using the PureLink RNA Mini Kit following the instruction from the manufacture. The integrity of RNA was confirmed by gel electrophoresis. Each RNA sample (10 µg) was treated with DNase (TURBO DNA-free) according to the manufacture’s instruction. DNase-treated RNA samples were used as templates to generate the first strand cDNA by the GoScript Reverse Transcription System. The cDNA products were diluted to 8 ng/µL and 2 µL was used as template for quantitative real-time PCR (qRT-PCR) using Power SYBR Green as described previously [32]. Primers for neighboring genes as well as the house-keeping gene *ACT1* are included in Supplemental Table S2. The relative levels of transcripts were quantified by ΔΔCt method as we previously described [33]. Three biological replicates were used. Statistical significance was determined using a 2-tailed *t* test.

### 2.5. Pulsed Field Gel Electrophoresis

Pulsed field gel electrophoresis (PFGE) was performed according to the procedures described previously [34,35]. Briefly, strains were grown in YPD liquid medium at 30 °C with shaking at 220 rpm for 20 h and then transferred to liquid YNB medium with 1 M NaCl at 30 °C with shaking at 220 rpm for an additional 24 h. Cells were washed twice with buffer (50 mM EDTA with 0.5 M NaCl, pH 8.0) and then kept on ice. Protoplasts were prepared by digestion with 10 mg/mL zymolase in solution buffer (10 mM KPO_4_, pH 7.5) as described previously [34]. The agarose plugs were prepared by mixing 0.5% low melting point agarose in 100 mM EDTA (pH 7.5) with protoplasts [36]. After solidifying the plugs, we incubated the plugs with solution (500 mM EDTA and 10 mM Tris, pH 7.5) at 37 °C for 12 h, followed by the addition of another solution (5% sarcosyl, 5 mg/mL proteinase K in 500 mM EDTA, pH 7.5) and incubated at 50 °C for 12 h. After washing the plugs with the buffer (50 mM EDTA, Tris 20 mM, pH 8.0), the plugs were stored at 4 °C. Chromosomal separations were carried out with a contour-clamped homogeneous electric field CHEF mapper XA apparatus (BioRad, Hercules, CA, USA) using the large cast (dimension ~14 × 20 cm) and switching time of (linear ramp) 120–360 s, 3.6 V/cm. We used 1% BioRad Megabase certified agarose in 0.5× TBE and the gel was run at 14 °C for 116–120 h. After running, the gel was stained by ethidium bromide (EB) for 30 min at room temperature. Images were obtained with the ChemStudio Imaging system (Analytik Jena, Jena, Germany).

### 2.6. Fluorescence Microscopy

Fluorescent microscopy was performed using a Zeiss Imager M2 microscope (Zeiss, Oberkochen, Germany). The indicated strains were cultured in liquid YPD medium overnight at 30 °C and washed with sterile ddH_2_O. Images were acquired with an AxioCam MRm camera and processed with Zen pro software. The fluorescence intensity of 30 individual cells from each strain imaged at 63× magnification was quantified using ZEN “Histo definition” quantification software application. Each cell and background were selected using the circular selection tool and the average fluorescence intensity within that circle was recorded. The fluorescence intensity of each cell and its background was measured. The relative fluorescence intensity of each cell was normalized by subtracting the fluorescence intensity of the cell’s background.

### 2.7. In Vitro Phenotypic Analysis

The indicated strains were cultured overnight in liquid YPD medium at 30 °C with 220 rpm. The cells were washed with ddH_2_O and adjusted to the same cell density (optical density at 600 nm [OD600] = 1.0), and then serially diluted. The serial dilutions of each strain (3 µL) were spotted onto various agar media for phenotypical analyses. To test thermotolerance of the indicated strains, cells were spotted onto YPD medium and incubated at 22 °C, 30 °C and 37 °C for 2 days. To analyze melanization, cells were spotted onto agar medium containing L-dihydroxyphenylalanine (L-DOPA) and incubated at 37 °C in the dark. To observe capsule production, cells were spotted onto RPMI agar medium and grown at 37 °C with 10% CO_2_. To test the ability of fungal cells to grow in cell wall stress and osmotic stresses, cells were grown on YPD medium supplemented with Congo Red (0.05%), NaCI (1.5 M), or KCI (1.5 M).

### 2.8. Phagocytosis Assay

Murine macrophage cells were used to evaluate phagocytosis as we previously described [37]. Mouse macrophage cell line J774A.1 (ATCC TIB-67TM) was acquired from the American Type Culture Collection and cultured in Dulbecco’s modified Eagle’s medium (DMEM) (catalog no. 30-2002) with 10% fetal bovine serum (FBS). Freshly grown J774A.1 cells were seeded in 24-well microtiter plates (2 × 10^5^ cells per well) and cultured at 37 °C with 5% CO_2_ for 24 h. Before the coculture, *C. gattii* cells (2 × 10^6^ cells per well) were opsonized with mouse serum for 30 min. The serum was collected from mice infected with H99 strain from a previous study [32].Then the old culture medium from macrophages was replaced with fresh DMEM medium containing opsonized *C. gattii* cells. After mixing, the cocultures were incubated at 37 °C with 5% CO_2_ for 2 h. Then cocultures were washed three times with warm Dulbecco’s phosphate-buffered saline (DPBS) to remove non-adherent cells. The macrophages were checked under an inverted microscope (Eclipse Ti; Nikon, Tokyo, Japan) and then lysed by chilled PBS with 0.1% Triton X-100. The cell suspension was collected and diluted, plated on the YNB medium, and incubated at 30 °C for 2 days for colony forming unit (CFU) enumeration.

## 3. Results

### 3.1. Identification of the Putative Safe Haven Regions in the Genome of C. gattii Reference Strain R265

*C. gattii* reference strain R265 was used to identify potential safe haven regions. R265 belongs to the VGII molecular type, which is predominant among clinical and environmental isolates from Vancouver Island and is responsible for the majority of cases of cryptococcosis caused by *C. gattii* [7,8,38]. Furthermore, its genome has been sequenced and annotated [39] and transcriptome data are available [26,40]. Lastly, congenic pair strains (AIR265**a** and AIR265α), which are genetically similar and differ primarily in the mating type locus, are only available in an R265 background [41,42]. Therefore, characterization of safe haven regions in this *C. gattii* reference strain would likely generate most impact in this field.

**Figure 1:**
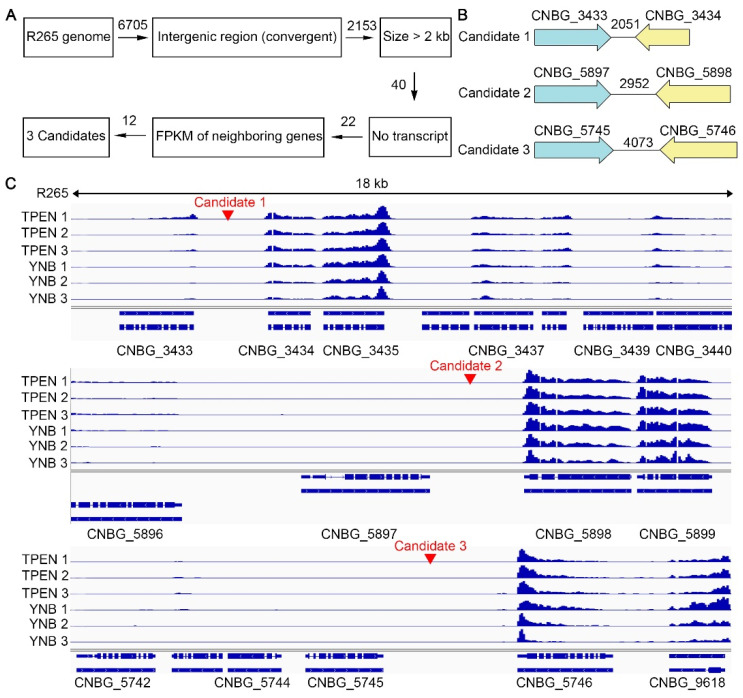
Identification of putative safe haven sites in *Cryptococcus gattii* R265. (**A**) Flow chart indicating the selection criteria for potential safe haven candidates. (**B**) Diagram of the three candidate regions including their neighboring genes and the size of intergenic regions. The arrows indicate the transcription direction of the genes. (**C**) A genome browser shot of the transcript profiles for the three potential safe haven candidates based on the published RNA-seq data of R265 cultured in YNB medium, with or without the zinc chelator TPEN [26]. The red arrows indicate the designed guide RNA target site used in this study.

The selection process for potential safe haven candidates is depicted in the flow chart (Figure 1A). 6705 intergenic regions in the genome of *C. gattii* reference strain R265 were identified and classified into three categories based on the relative transcriptional orientations of the two neighboring genes: convergent (terminator–terminator or tail–tail), divergent (promoter–promoter or head–head), and tandem (promoter–terminator or head–tail). A total of 40 convergent intergenic regions (out of 2153) larger than 2 kb were selected to minimize potential disruption of any nearby promoters and terminators. We examined transcript profiles at these 40 intergenic regions and their neighboring genes based on the RNA-seq data of R265 cultured in YNB medium with or without the addition of the zinc chelator TPEN [26]. The median transcript levels of *C. gattii* genes in these data sets generally lie somewhere between 10 and 20 FPKM (fragments per kilobase of transcript per million). Examining the transcript profiles of these regions allowed us to exclude 18 intergenic regions where transcripts were detected. To ensure safe haven regions are accessible for the expression of the inserted genes, 10 out of remaining 22 intergenic regions where both neighboring genes showed no transcripts were excluded because these regions could potentially be in a heterochromatin state, repressed for transcription. Eventually, we picked 3 out of remaining 12 intergenic regions for further characterization (Figure 1B,C): Candidate 1—the 2051 bp intergenic region between CNBG_3433 and CNBG_3434 on chromosome 1; Candidate 2—the 2952 bp intergenic region between CNBG_5897 and CNBG_5898 on chromosome 4; and Candidate 3—the 4073 bp intergenic region between CNBG_5745 and CNBG_5746 on chromosome 4.

### 3.2. Insertion of Foreign DNA into the Candidate 1 or the Candidate 2 Site Has No Significant Impact on the Expression of the Neighboring Genes

**Figure 2:**
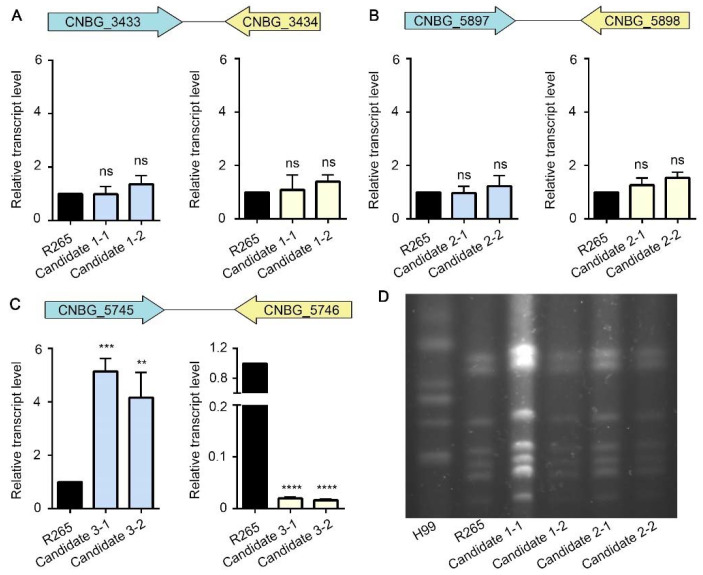
DNA insertion in the candidate 1 site or the candidate 2 site has no significant impact on the expression of their neighboring genes and chromosomal rearrangement. (**A**–**C**) The relative transcript levels of the genes neighboring the three intergenic safe haven regions in the indicated strains were measured by RT-PCR. The transcript level of house-keeping gene *ACT1* was used as the internal control in every sample. The transcript level of each gene was compared to that in the wild-type R265, which was set to 1 for normalization. The experiments were performed in three independent biological replicates. Statistical significance was determined using a 2-tailed *t* test: ns, not significant, **, *p* < 0.01; ***, *p* < 0.001; ****, *p* < 0.0001. (**D**) Image of a PFGE gel of the genomic DNA preparations from the indicated strains.

Our selection criteria for safe haven candidates help minimize the chance of disrupting the neighboring genes physically or altering their expression due to the integrated foreign DNA fragment. To verify that experimentally, we transformed *C. gattii* R265 with a construct carrying an mNeonGreen fluorescence reporter gene and a selection marker and targeted the integration to the candidate safe haven sites using the transient CRISPR-Cas9 expression (TRACE) system that we established previously [13,32]. We then randomly picked transformants and screened for correct integration into the three safe haven candidate sites by diagnostic PCR (Figure S1). We selected two transformants that integrated correctly at each candidate safe haven site for further characterization (Figure S1). To examine if transcript levels of genes bordering the candidate safe haven sites are altered by the integration of foreign DNA in these transformants, we measured the transcript levels of CNBG_3433 and CNBG_3434 in the two selected transformants for candidate 1, of CNBG_5897 and CNBG_5898 in the two selected transformants for candidate 2, and of CNBG_5745 and CNBG_5746 in the two selected transformants for candidate 3. The transcript level of CNBG_3433 and CNBG_3434 in the selected transformants for candidate 1 site showed no apparent difference compared to those of the parental strain (Figure 2A). Similarly, no significant differences in expression of CNBG_5897 and CNBG_5898 were observed in the selected transformants for candidate 2 site compared to the parental strain R265 (Figure 2B). However, the transcript level of CNBG_5745 was drastically higher and the transcript level of CNBG_5746 was markedly lower in the selected transformants for candidate 3 site compared to the parental strain (Figure 2C). Therefore, we removed the candidate 3 site for further analysis. We used pulsed field gel electrophoresis (PFGE) for chromosomal analysis to examine if the insertion of foreign DNA fragment into the candidate 1 or the candidate 2 site could cause any gross chromosomal rearrangement in these transformants. As shown in Figure 2D, the pattern of chromosomes on PFGE gel of these strains was indistinguishable from that of the wild-type R265 strain. As expected, the pattern of chromosomes of *C. gattii* R265 differs from that of *C. neoformans* H99 (Figure 2D). These data suggest that DNA insertion into candidate 1 and candidate 2 sites does not influence the transcript level of the neighboring genes and does not cause gross chromosomal rearrangement.

### 3.3. Genes Inserted in the Candidate 1 and Candidate 2 Sites Are Expressed Irrespective of the Insertion Direction

**Figure 3:**
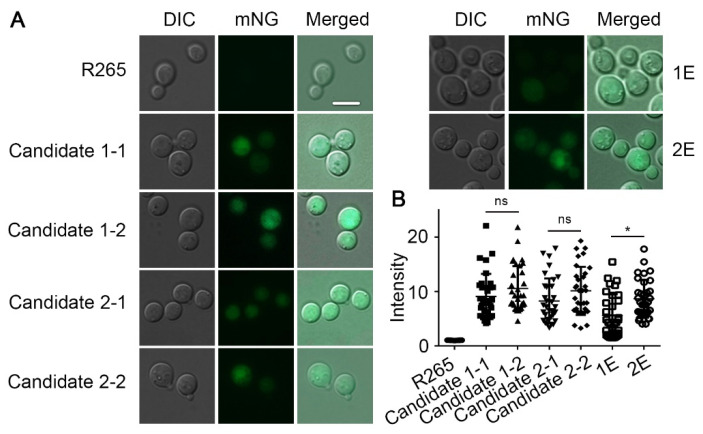
The mNeonGreen integrated into either the candidate 1 site or the candidate 2 site is expressed at a similar level irrespective of the insertion direction. (**A**) Images of the selected transformants and the wild-type strain R265 were examined microscopically under DIC and the GFP filter. Scale Bar, 5 μm. (**B**) Quantification of the fluorescence intensity in the indicated isolates by measuring the fluorescence as detailed in the method using Zeiss ZEN 3.0 software. Statistical significance was determined using a one-way ANOVA statistical analysis. * *p* < 0.05.

As mentioned earlier, we need the safe haven sites to be permissive for gene expression. As we have the green fluorescent protein gene in the construct, we verified their expression in the transformants by examining their fluorescence. Because we did not include any homologous arms to the safe haven candidate sites in our construct, the DNA fragment could insert into the candidate intergenic regions either in the forward or the reverse direction. Indeed, among the transformants we randomly selected, we had transformants with the construct inserted into the two candidate regions in either direction based on diagnostic PCR (Figure S1). As shown in Figure S1, the different sizes of PCR products by the primer set could help us confirm the orientation of transformants. We also have transformants with the construct being inserted into the genome ectopically. We measured the fluorescence intensity of the selected transformants of candidate 1 and candidate 2 sites in the forward (candidate 1-1 and candidate 2-2) and the reverse (candidate 1-2 and candidate 2-1) orientation. We also included transformants with ectopic insertions (1E and 2E). As shown in Figure 3 and Figure S2, the fluorescence intensity among the transformants inserted in the candidate 1 or candidate 2 sites was comparable. By contrast, the ectopic insertion 1E showed lower fluorescent intensity compared to 2E. Moreover, there were no significant differences between the forward and reverse insertions in both candidate 1 and candidate 2 transformants (Figure 3 and Figure S2). These results indicate that the expression level of mNG integrated at the candidate 1 or the candidate 2 site is comparable and is independent of the insertion direction.

### 3.4. Insertion of the Foreign DNA Construct in the Candidate 1 and Candidate 2 Sites Has No Significant Impact on Growth of C. gattii, Its Response to Various Stresses, or Its Ability to Undergo Sexual Reproduction

**Figure 4:**
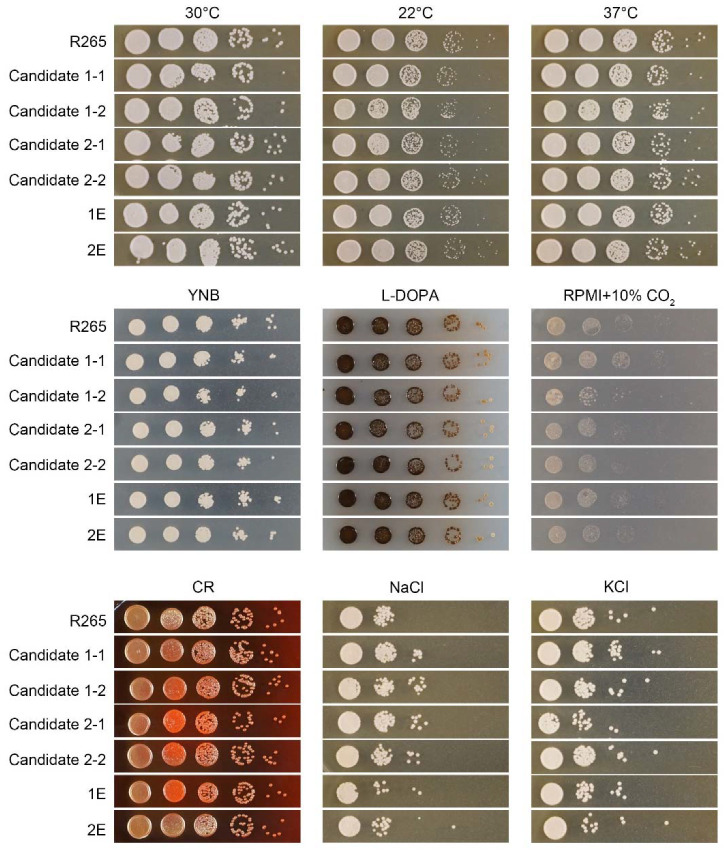
Insertion of the foreign DNA construct in the candidate 1 site or the candidate 2 site has no significant impact on growth of *C. gattii* or its response to various stresses. Cells of the indicated strains were tested for thermotolerance using a spotting assay with serial dilutions on YPD medium. Cells were incubated at 22 °C, 30 °C, and 37 °C for 2 days. The same serial dilutions of these strains were also tested for melaninization on L-Dopa medium and capsule formation on RPMI medium with 10% CO_2_ (see Figure S3 for Indian ink staining for capsule). Cells were cultured on YPD with Congo Red (0.05%), NaCl (1.5 mM), or KCl (1.5 mM) to test their tolerance of cell wall and osmotic stresses.

For an ideal safe haven region, the insertion of DNA itself in this region should not alter the phenotypes. Here, we are not considering the cases where the products of the inserted DNA fragment will alter fungal biology (e.g., resistance to the selection drug and the expression of mNG). To test the impact of DNA insertion in the candidate safe haven sites, we examined various phenotypes of the transformants of candidate 1 and candidate 2, and compared them to the wild-type strain R265. As shown in Figure 4, these strains showed no obvious difference from the wild-type strain when grown at 22 °C, 30 °C, and 37 °C, indicating that the DNA insertion in the candidate 1 or the candidate 2 safe haven sites does not affect cryptococcal thermotolerance. Moreover, the growth of these strains was comparable to R265 on the minimal YNB medium, suggesting that DNA integration at the candidate sites does not cause them to become auxotrophic or affect growth rate under nutrient-limiting conditions. Likewise, no apparent difference in melanization or capsulation, compared to R265 was observed when these strains were cultured on the L-Dopa or RPMI medium (Figure 4 and Figure S3). We also tested these strains for their susceptibility to cell wall stress using Congo Red, and osmotic stresses using NaCl and KCl. Again, these strains behaved similarly to the wild-type strain under the tested conditions. Collectively, these results indicate that insertion into either the candidate 1 or the candidate 2 safe haven sites does not affect cryptococcal growth under different stresses.

**Figure 5:**
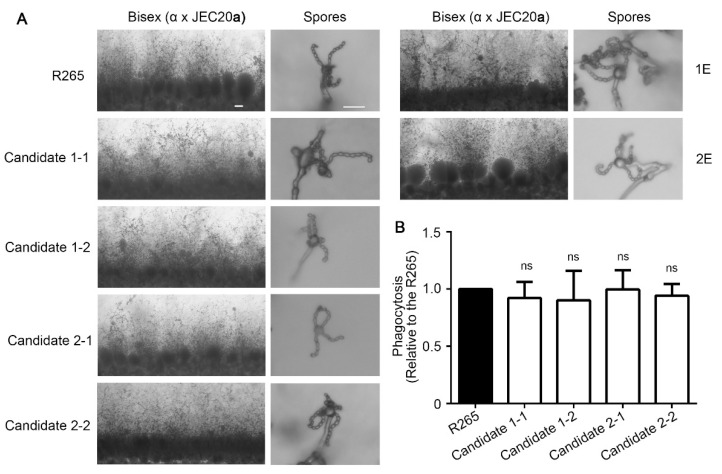
DNA insertion in the candidate 1 or the candidate 2 site has no apparent impact on sexual reproduction and phagocytosis of *C. gattii*. (**A**) The indicated strains were diluted to equal concentrations and cultured on V8 agar medium with the mating partner JEC20**a** at the same cell density at 22 °C in the dark for 14 days. Images of the edges of the mating colonies (scale bar: 100 μm) and the fruiting bodies (scale bar: 10 μm) were taken. (**B**) The indicated strains were opsonized with mouse serum for 30 min before incubation with murine J774A.1 macrophage cells for two hours. Non-adherent *C. gattii* cells were washed with DPBS. Phagocytosed and adherent *C. gattii* cells were then released from lysed macrophages and plated on YNB medium for CFU counting. The percentage of phagocytosis was calculated by the number of recovered CFUs versus the starting number of *cryptococcus* cells. The phagocytosis of R265 was set to 1 for normalization. The experiments were performed in three independent biological replicates. Statistical significance was determined using a 2-tailed *t* test. ns, not significant.

We also tested these strains for their ability to undergo **a**-α bisexual mating by mixing these α strains with the JEC20**a** tester strain and co-culturing them on V8 juice agar medium. During mating, α and **a** cells fuse and form dikaryotic zygotes. The zygotes then transition to hyphal growth and aerial hyphal tips eventually form club-shaped basidial heads where meiosis and sporulation occur, forming four chains of basidiospores [2]. As expected, wild-type R265 successfully mated with JEC20**a** (Figure 5A) and we did not observe any significant differences in bisexual mating filamentation or sporulation in any of these strains (Figure 5A). Thus, integration of DNA at the candidate 1 or candidate 2 sites does not affect sexual reproduction.

To examine whether the insertion at these two candidate sites would influence *C. gattii* interaction with host cells, we tested phagocytosis of these strains by mammalian macrophage cells. Here, we co-cultivated J774A.1 murine macrophage cells and the indicated cryptococcal strains for 2 h. As shown in Figure 5B, the transformants of the candidate 1 and candidate 2 sites showed a similar level of phagocytosis compared to the wild-type strain, implying that the insertion of DNA at the putative safe haven regions had no apparent impact on phagocytosis of *C. gattii*. Taken together, the candidate 1 site and the candidate 2 site appeared to be “safe” regions to insert DNA and thus we named them CgSH1 and CgSH2.

### 3.5. A Similar Intergenic Region Exists in Strains of Other Molecular Types within the C. gattii Species Complex

**Figure 6:**
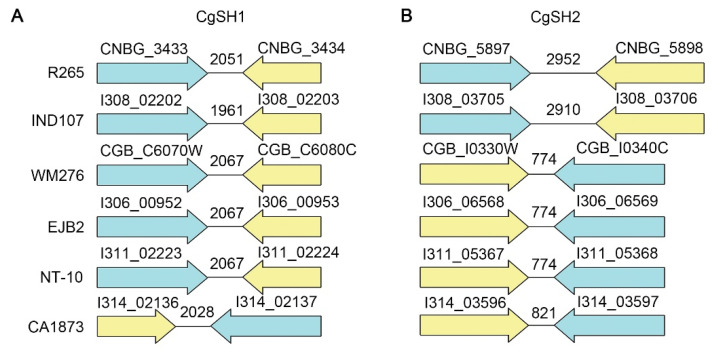
Comparison of intergenic regions in six other *C. gattii* strains that represent all four molecular types. The diagrams show the organization of the CgSH1 (**A**) and CgSH2 (**B**) equivalent intergenic regions and neighboring genes in the six indicated *C. gattii* strains that represent four molecular types.

Considering the evolutionary separation of the four molecular types within the *C. gattii* species complex (VGI to VGIV), it is not surprising that there are considerable genome differences among them, including chromosomal rearrangements and nucleotide sequence divergence [27,33]. To determine if CgSH1 and CgSH2 identified in R265 could be useful in other strains within the *C. gattii* species complex, we analyzed the genome sequences of six *C. gattii* strains that encompass all four molecular types: VGI (WM276, NT-10 and EJB2), VGII (R265), VGIII (CA1873), and VGIV (IND107). Based on homology analyses of the neighboring genes of CgSH1 and CgSH2 in R265 and these other strains (CNBG_3433-CNBG_3434, and CNBG_5897-CNBG_5898), we found that both gene pairs remain as neighbors in all these strains (Figure 6). However, the size of their intergenic regions differs among the six strains. Compared to CgSH2 in R265, the intergenic regions in other five strains were smaller (774 bp or 821 bp), with the exception of CA1873 (2910 bp) (Figure 6B), implying that the CgSH2 is suitable to use in R265 (VGII) and CA1873 (VGIII) and might be problematic in other strains. By contrast, despite the differences in DNA sequence of the intergenic region for CgSH1 among the six strains (as expected for intergenic region), the size of CgSH1 was similar among these strains, ranging from 1961 bp to 2061 bp (Figure 6A). Thus, CgSH1 might be a safe haven site that is universally suitable in all species in the *C. gattii* species complex.

## 4. Discussion

In this work, we screened the genomes of the *C. gattii* serotype B reference strain R265 and identified two gene free regions CgSH1 and CgSH2 that can serve as safe haven sites for DNA integration. In addition to the information of genome sequence and gene annotations, we capitalized the publicly available RNA-seq data to minimize the risk of selecting unrecognized transcribed regions or potentially repressed heterochromatin regions [26,39]. Our results showed that an inserted fluorescent reporter gene in the CgSH1 or CgSH2 region can be expressed efficiently, and the insertion itself does not affect the expression of the neighboring genes. Based on the examination of transformants with the DNA integrated at these sites, we demonstrated that DNA insertion into CgSH1 or CgSH2 has no apparent impact on cryptococcal growth, the typical virulence traits, stress responses, and phagocytosis. The *C. gattii* species complex contains at least five recognized species or four molecular types [38]. However, the acceptance of these subspecies has been controversial [43,44]. For simplicity, we referred them all as *C. gattii* in this study. According to the alignment of CgSH1 and CgSH2 based on neighboring genes in multiple strains representing all four molecular types of the *C. gattii* species complex, similar gene free regions exist in other *C. gattii* strains. In particular, the intergenic region represented by CgSH1 in all strains examined are of a reasonable size, suggesting that CgSH1 might be useful for integrating foreign DNA fragments in most, if not all, strains of the *C. gattii* species complex. Taken together, these newly identified CgSH1 and CgSH2 regions provide a good tool for genetic manipulation in *C. gattii*.

## Data Availability

Not applicable.

## Supplementary Materials

The following supporting information can be downloaded at: https://www.mdpi.com/article/10.3390/jof8020178/s1, Figure S1: Confirmation of the selected transformants by diagnostic PCR; Figure S2: The mNeonGreen integrated into either the candidate 1 site or the candidate 2 site is expressed at a similar level irrespective of the insertion direction; Figure S3: DNA insertion in the candidate 1 or the candidate 2 site has no significant impact on capsulation; Table S1: Strains used in this work [45]; Table S2: Primers used in this work.
