# Supplementary material for: Identification and Characterization of an Intergenic “Safe Haven” Region in Human Fungal Pathogen Cryptococcus gattii
Source: J Fungi (Basel). 2022 Feb 11;8(2):178. doi: 10.3390/jof8020178 (PMC8874978; doi:10.3390/jof8020178)
Supplement: Supplementary file 1 [file jof-08-00178-s001.zip › jof-1587017-supplementary.pdf]

**Identification and characterization of an intergenic "safe haven" region in human fungal pathogen *Cryptococcus gattii***

**Yeqi Li[1], Tuyetnhu Pham[2], Xiaofeng Xie[1] and Xiaorong Lin[1,2]***

1 Department of Microbiology, University of Georgia, Athens, GA 30602, USA
2 Department of Plant Biology, University of Georgia, Athens, GA 30602, USA
* Correspondence: xiaorong.lin@uga.edu

Supplemental Table S1. Strains used in this work

| Strain name | Background | Genotype | Reference |
|---|---|---|---|
| R265 | R265 | MAT alpha | [1] |
| YL03 | R265 | MAT alpha, $P_{TEF1}$-mNG-HYG in SH3 F-mNG iso 1 | This study |
| YL04 | R265 | MAT alpha, $P_{TEF1}$-mNG-HYG in SH3 F-mNG iso 2 | This study |
| YL07 | R265 | MAT alpha, $P_{TEF1}$-mNG-HYG | This study |
| YL08 | R265 | MAT alpha, $P_{TEF1}$-mNG-HYG | This study |
| YL20 | R265 | MAT alpha, $P_{TEF1}$-mNG-HYG in SH2 R-mNG iso 1 | This study |
| YL21 | R265 | MAT alpha, $P_{TEF1}$-mNG-HYG in SH2 F-mNG iso 2 | This study |
| YL23 | R265 | MAT alpha, $P_{TEF1}$-mNG-HYG in SH1 F-mNG iso 1 | This study |
| YL24 | R265 | MAT alpha, $P_{TEF1}$-mNG-HYG in SH1 R-mNG iso 2 | This study |

Supplemental Table S2. Primers used in this work

| Primer name | Sequence 5'-3' | Comment |
|---|---|---|
| Linlab4627 | ggctcaaagagcagatcaatg | U6 promoter FL_F |
| Linlab4628 | cctctgacacatgcagctcc | sgRNA terminator nested |
| Linlab4478 | GGTGACGCTGTGAGAGTGG | Cas9-F |
| Linlab4433 | GGGCCCCTCTTCACGTGG | Cas9-R |
| Linlab8004 | TCCATCACACTGGCGGCCGCAGGAGCATGATCCGATACAT | TEF1-promoter-F |
| Linlab8005 | TTTTTTGTCCATTTTTGGCCGGCCTTTGAAGTTTTCTGTGGAGA | TEF1-promoter-R |
| Linlab3779 | CCAATGCATATGATAAGAGTCGTCC | Inside of TEF promoter |
| Linlab8122 | GGAGAAGTTCAAGCGAGAAGCAACAGTATACCCTGCCGGTG | SH1-sgRNA-U6promoter |
| Linlab8123 | GCTTCTCGCTTGAACTTCTCCGTTTTAGAGCTAGAAATAGCAAGTT | SH1-sgRNA-scaffold |
| Linlab8147 | GCAACTGCCTTCTGAAGC | SH1-self-F |
| Linlab8148 | TGAGGTAAATAGAGATGG | SH1-self-R |
| Linlab8150 | GGAGAAGTTCAAGCGAGAAGCAACAGTATACCCTGCCGGTG | SH2-sgRNA-U6promoter |
| Linlab8151 | GCTTCTCGCTTGAACTTCTCCGTTTTAGAGCTAGAAATAGCAAGTT | SH2-sgRNA-scaffold |
| Linlab8152 | TGAGGAACATCTGGGTGG | SH2-self-F |
| Linlab8153 | CGTCTCCTTCAGCTCACT | SH2-self-R |
| Linlab8050 | TGTCTCATCTGCTGCGCCATAACAGTATACCCTGCCGGTG | SH3-sgRNA-U6promoter |
| Linlab8051 | ATGGCGCAGCAGATGAGACAGTTTTAGAGCTAGAAATAGCAAGTT | SH3-sgRNA-scaffold |
| Linlab8068 | GTTGACACTGGCGCTGGG | SH3-self-F |
| Linlab8069 | GAGGGACCTTGCAACGGA | SH3-self-R |
| Linlab8093 | GTATTGCCGACCGCATGCAA | RT-ACT1 F |
| Linlab8094 | TCGCTCTTCGCGATCCACAT | RT-ACT1 R |
| Linlab8095 | TGACTCTCGAAGCCGTGGAC | RT-CNBG_5745 F |
| Linlab8096 | CATCGTCCTCCTGCACCGAT | RT-CNBG_5745 R |
| Linlab8097 | TCCGGCGCCAACTGGATTAT | RT-CNBG_5746 F |
| Linlab8098 | ACCGACGACACAGAAGCACA | RT-CNBG_5746 R |
| Linlab8099 | ATCGTTGCGCAGGTTGGATT | RT-CNBG_3433 F |

| | | |
|---|---|---|
| Linlab8100 | CCACACCGGAGTCCCTAACA | RT-CNBG_3433 R |
| Linlab8101 | GCCAGGATTTCACGGAAGCA | RT-CNBG_3434 F |
| Linlab8102 | CTTCTACGATGCCGCCACTG | RT-CNBG_3434 R |
| Linlab8160 | ACGGCTTTGCCCGTCAATTC | RT-CNBG_5897 F |
| Linlab8161 | AACCCTTCCCTTTCCGCCAA | RT-CNBG_5897 R |
| Linlab8162 | GTGGAGATCGCTGGGCTCTT | RT-CNBG_5898 F |
| Linlab8163 | CTAGGACGGGCTTCGATGGG | RT-CNBG_5898 R |

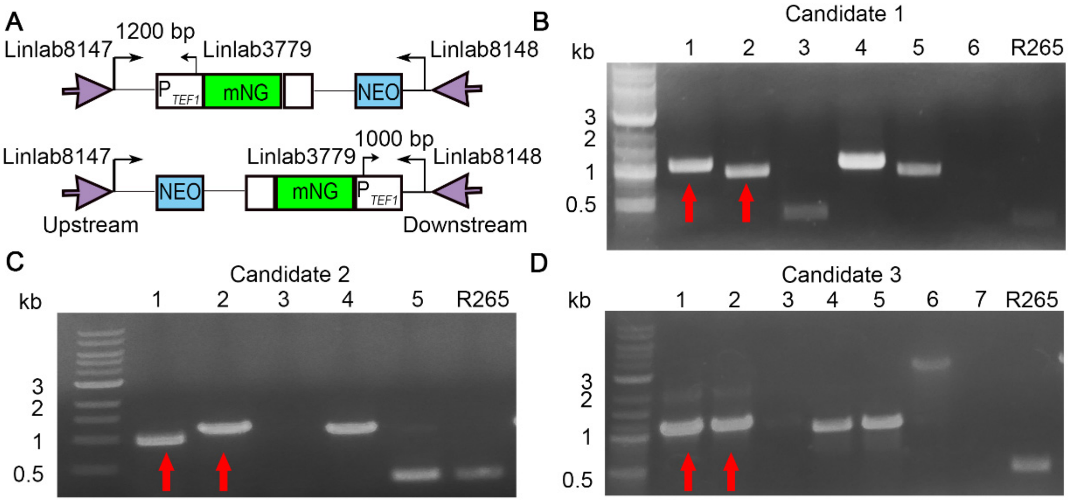


**Figure S1**. Confirmation of the selected transformants by diagnostic PCR.

(A) The diagrams for the diagnostic PCR of the selected transformants to examine the insertion in the candidates and the orientation of the insertion. For candidate 1, the primers Linlab3779/Linlab8147 will amplify 1200 bp fragment when the orientation of the insertion is forward. When the orientation of the insertion is reverse, primers Linlab3779/Linlab8148 will amplify 1000 bp fragment. The size of wild-type R265 was approximately 500 bp. For candidate 2 and candidate 3, primer set Linlab3779/Linlab8152/Linlab8153 and Linlab3779/Linlab8068/Linlab8069 were used. The purple arrows mean the neighboring genes of insertion sites. The diagnostic PCR of transformants for the candidate 1 site (B), the candidate 2 site (C), and the candidate 3 site (D) showed three different sizes. The transformants indicated by red arrows were selected for further characterization.

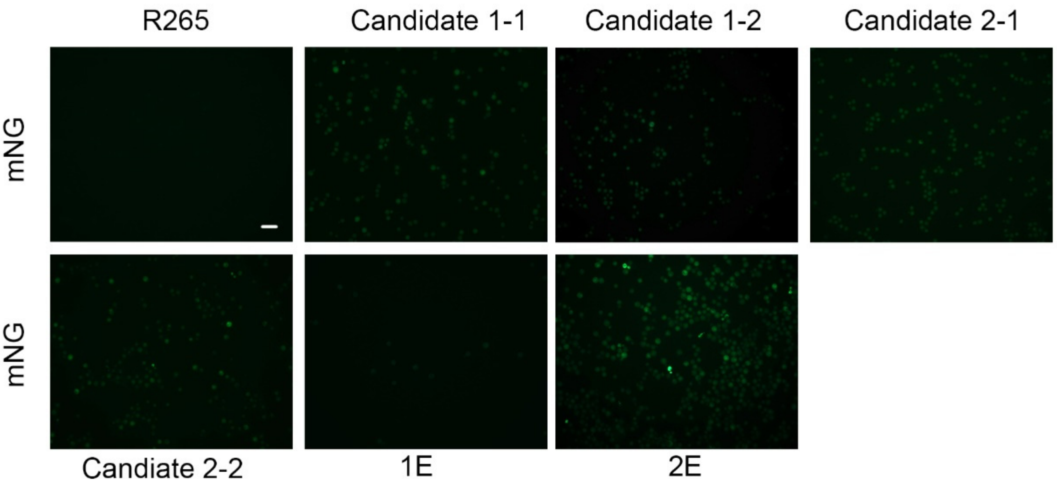


**Figure S2. The mNeonGreen integrated into either the candidate 1 site or the candidate 2 site is expressed at a similar level irrespective of the insertion direction.**
The selected transformants and R265 were cultured in liquid YPD medium overnight at 30°C with 220 rpm. Then the cells were washed with $ddH_2O$ and images of the selected transformants and the wild-type strain R265 were examined microscopically under the GFP filter. Scale Bar, 5 μm.

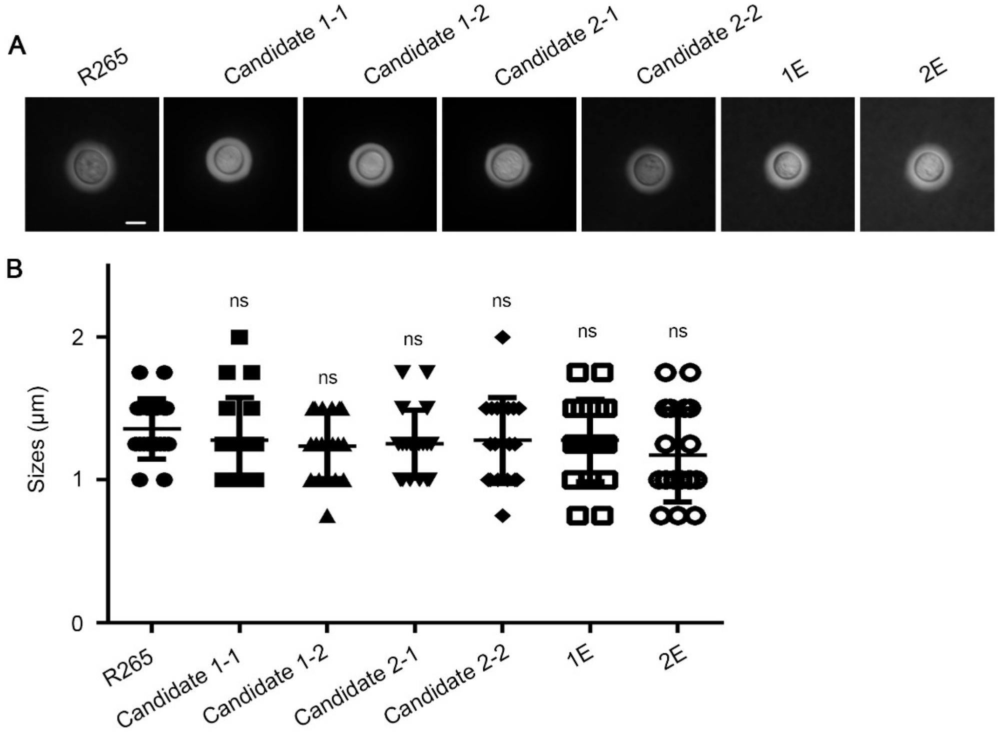


**Figure S3. DNA insertion in the candidate 1 or the candidate 2 site has no significant impact on capsulation.**
Cells were cultured on RPMI medium at 37°C with 10% $CO_2$ for 3 days. Capsule of the

indicated strain was visualized by counterstaining with India ink (A) and quantified by using the Zeiss Zen 3.0 (B).


1. Kidd, S.E.; Hagen, F.; Tscharke, R.L.; Huynh, M.; Bartlett, K.H.; Fyfe, M.; Macdougall, L.; Boekhout, T.; Kwon-Chung, K.J.; Meyer, W. A rare genotype of *Cryptococcus gattii* caused the cryptococcosis outbreak on Vancouver Island (British Columbia, Canada). *Proc Natl Acad Sci U S A* **2004**, *101*, 17258-17263, doi:10.1073/pnas.0402981101.
